# The phase I trial of preoperative short-course chemoradiotherapy with S-1/irinotecan and short-course radiation in locally advanced lower rectal cancer (SHOWTIME study)

**DOI:** 10.1186/s13014-026-02872-3

**Published:** 2026-06-10

**Authors:** Ken Kojo, Toshimichi Tanaka, Takahiro Yamanashi, Hirohisa Miura, Keigo Yokoi, Keita Kojima, Kazuko Yokota, Shogo Kawakami, Hiromichi Ishiyama, Keishi Yamashita, Yusuke Kumamoto, Naoki Hiki, Takeo Sato, Takeshi Naitoh

**Affiliations:** 1 Department of Lower Gastrointestinal Surgery, Kitasato University School of Medicine, 1-15-1, Kitasato, Minami Ward, Sagamihara, Kanagawa 252-0329 Japan; 2 Department of Radiation Oncology, Kitasato University School of Medicine, 1-15-1 Kitasato, Minami Ward, Sagamihara, Kanagawa 252-0329 Japan; 3 Division of Advanced Surgical Oncology, Research and Development Center for New Medical Frontiers, Kitasato University School of Medicine, 1-15-1 Kitasato, Minami Ward, Sagamihara, Kanagawa 252-0329 Japan; 4 Department of General-Pediatric-Hepatobiliary Pancreatic Surgery, Kitasato University School of Medicine, 1-15-1 Kitasato, Minami Ward, Sagamihara, Kanagawa 252-0329 Japan; 5 Department of Upper Gastrointestinal Surgery, Kitasato University School of Medicine, 1-15-1 Kitasato, Minami Ward, Sagamihara, Kanagawa 252-0329 Japan; 6 Research and Development Center for Medical Education, Department of Clinical Skills Education, Kitasato University School of Medicine, 1-15-1 Kitasato, Minami Ward, Sagamihara, Kanagawa 252-0329 Japan

**Keywords:** Advanced rectal cancer, Chemoradiotherapy, Total neoadjuvant therapy

## Abstract

**Background:**

A phase I study was conducted to determine the optimal dose of irinotecan in short-course chemoradiotherapy (SCCRT) using S-1/irinotecan and short-course radiotherapy (SCRT) for the development of a new total neoadjuvant therapy (TNT) regimen that is safe, effective, and has a low patient burden for locally advanced rectal cancer (LARC).

**Methods:**

The objective of this study was to ascertain the maximum tolerated dose (MTD) and the recommended dose (RD) of irinotecan in SCCRT for locally advanced rectal cancer. The assessment of dose-limiting toxicity (DLT) was conducted over a 14-day period following treatment administration. The patient received a 14-day course of chemotherapy, with S-1 administered from the first to the fifth day and from the eighth to the twelfth day. Irinotecan was administered on the first and eighth days of treatment. The radiotherapy regimen was initiated with a total dose of 25 Gy, administered in five fractions from the eighth to twelfth day.

**Results:**

Fifteen patients received SCRT in conjunction with S-1 and escalating doses of irinotecan (40–60 mg/m²). All patients completed the SCCRT protocol. At a dose of 60 mg/m², DLT occurred in all patients, thereby establishing this dosage as the MTD. At a dose of 50 mg/m², DLT was observed in two of six patients, thereby establishing this dose as the RD. The most prevalent Grade ≥ 3 adverse event was diarrhea. All patients received CapeOX-based consolidation chemotherapy. Among the 11 surgical cases, the R0 resection rate was 100%, with a pathological complete response in 27.3% and downstaging in 90.9%. Three patients demonstrated a complete clinical response and underwent non-operative management.

**Conclusion:**

The safety and feasibility of administering SCCRT with S-1/irinotecan at a 50 mg/m² irinotecan dose have been demonstrated. These findings support further investigation of this regimen in a TNT setting for rectal cancer.

**Trial registration:**

The present study was duly registered with the Japan Clinical Trials Registry (jRCT s031210461). URL: https://jrct.mhlw.go.jp/re.

**Supplementary Information:**

The online version contains supplementary material available at 10.1186/s13014-026-02872-3.

## Background

Rectal cancer accounts for approximately 30% of all colorectal cancers, and lower rectal cancer poses a significant therapeutic challenge due to its propensity for local recurrence and systemic metastasis [1, 2]. While preoperative chemoradiotherapy (CRT) followed by total mesorectal excision (TME) is the established standard, the paradigm has shifted toward Total Neoadjuvant Therapy (TNT) to address the historically poor adherence to postoperative adjuvant chemotherapy [3, 4].

Several landmark Phase 3 trials have underscored the clinical utility of TNT. In the RAPIDO trial, TNT utilizing short-course radiotherapy (SCRT) followed by systemic chemotherapy demonstrated a significant reduction in the probability of disease-related treatment failure (DrTF) compared to conventional CRT [5]. Similarly, the STELLAR trial reported that SCRT-based TNT was noninferior for overall survival and superior for disease-free survival (DFS) compared with standard CRT [6]. Beyond oncological efficacy, SCRT-based TNT is increasingly recognized for its health-economic advantages, notably the significant reduction in hospital visits [7]. This is particularly relevant in the context of major infectious disease outbreaks, where minimizing hospital exposure is critical for reducing the risk of nosocomial transmission and alleviating the burden on healthcare systems. At the same time, this method offers a significant socio-economic advantage by reducing both the direct and indirect financial burdens on patients caused by frequent hospital visits.

Our group previously demonstrated the efficacy and safety of combining S-1 and irinotecan with long-term radiotherapy in the SAMRAI trial [8]. The findings indicated that the combination of chemoradiotherapy with S-1/irinotecan was both effective and relatively straightforward to manage in terms of adverse effects when used with long-term radiotherapy. However, this regimen has not yet been validated in a short-course setting.

Combining the potent S-1/irinotecan doublet with SCRT (short-course chemoradiotherapy; SCCRT) could optimize preoperative treatment intensity while preserving the logistical advantages of SCRT. Therefore, we designed the SHOWTIME study to evaluate a novel TNT regimen that uses SCCRT followed by consolidation chemotherapy. Given the limited evidence on the concurrent use of S-1/irinotecan and SCRT, the primary objective of this study was to assess the safety and feasibility of SCCRT as a foundational step toward evaluating the complete TNT protocol.

## Patients and methods

### Trial design and participants

**Fig. 1:**
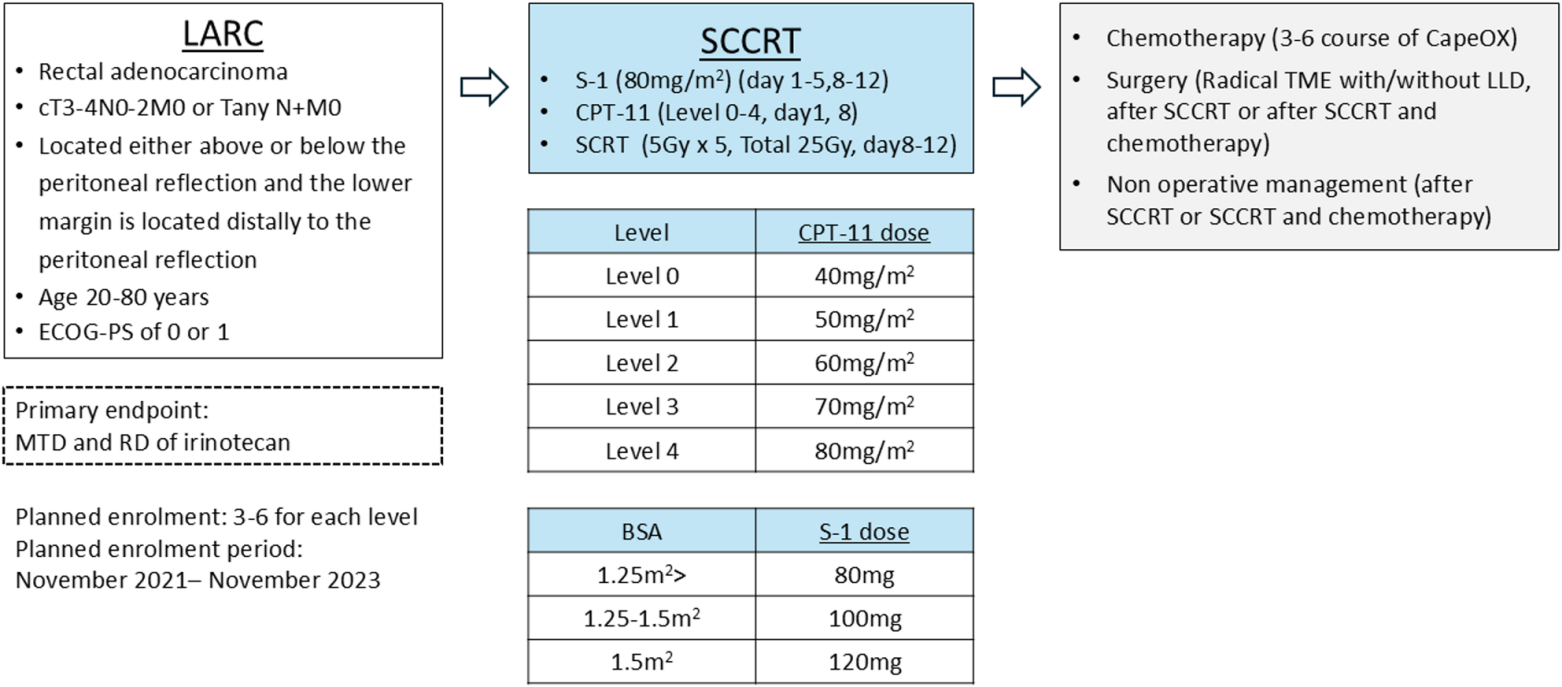
Study design. LARC, lower rectal cancer carcinoma; ECOG-PS, Eastern Cooperative Oncology Group performance status; MTD, maximum tolerated dose; RD, recommended dose; SCCRT, short-course chemoradiotherapy; SCRT, short-course radiotherapy; TME, total mesorectal excision; LLD, lateral lymph node dissection

The present study was a prospective, single-center, open-label, Phase I study (Fig. 1) and was properly registered with the Japan Clinical Trials Registry (jRCT s031210461).

The eligibility criteria were as follows: histologically confirmed rectal cancer (adenocarcinoma) cases that had not received any prior treatment, and that satisfied all of the following criteria based on imaging and hematological and physiological tests performed within 28 days before registration: Eligible cases were clinically diagnosed as cT3-4cN0cM0 or cTanycN+cM0 according to the Union for International Cancer Control (UICC) TNM classification, 8th edition [9]. The primary tumor is located either above or below the peritoneal reflection. The tumor margin is located at the lowermost extent, distal to the peritoneal reflection. The patient is between 20 and 80 years of age at the time of registration, with an Eastern Cooperative Oncology Group Performance Status (ECOG-PS) of 0 or 1, and no severe dysfunction of major organs (e.g., bone marrow, liver, kidneys). The following criteria were used to determine exclusion from the study: Active multiple cancers (i.e., simultaneous or metachronous cancers with a disease-free period of ≤ 5 years), the administration of flucytosine or atazanavir sulfate, the genetic phenotype UGT1A1*6/*6, UGT1A1*28/*28, and both heterozygotes (UGT1A1*6 /*28) were considered. Serious complications may include intestinal paralysis, intestinal obstruction, active or history of interstitial pneumonia or pulmonary fibrosis, poorly controlled diabetes, hypertension, heart failure, renal failure, hepatic failure, and other conditions. The patient exhibits signs of pleural effusion, ascites, or diarrhea (watery stools) requiring treatment, and is HBs antigen positive. The complete inclusion and exclusion criteria are provided in data S1.

### Treatment

Preoperative radiotherapy was administered concurrently with the second cycle of S-1/irinotecan. A total of 25 Gy in 5 fractions (5 Gy per fraction) was delivered over five consecutive days using 10-MV (or higher) photon beams. The treatment was completed within 10 days to allow for potential technical delays or holidays. Radiation was delivered via three-dimensional conformal radiation therapy (3D-CRT) using either a 3-field (posterior and bilateral) or 4-field (anteroposterior and bilateral) technique, with all fields treated in each session. The Gross Tumor Volume (GTV) included the primary tumor and radiologically suspicious lymph nodes, including suspected involvement of adjacent organs. The Clinical Target Volume (CTV) comprised the GTV with a 10-mm margin, the entire mesorectum, and the internal iliac and obturator lymph node regions. The Planning Target Volume (PTV) was defined by adding a 5-10-mm margin to the CTV to account for internal organ motion and set up uncertainties. Standard field borders for the PTV were defined as follows: the superior border at the L5-S1 junction; the inferior border at the lower edge of the ischiatic tuberosity (typically 3–4 cm below the distal tumor edge); the lateral borders at the pelvic sidewalls; the anterior border at the posterior edge of the pubic symphysis; and the posterior border at the posterior edge of the sacrum. We spared the anal skin unless the distal tumor margin necessitated its inclusion. Treatment planning was performed using a CT simulator, with patients in either supine or prone position (with or without a belly board). Dose distributions were calculated to ensure that the PTV was covered by 90%–110% of the prescribed dose at the isocenter. Heterogeneity corrections were applied for Monitor Unit (MU) calculations, except in cases of significant bowel gas or residual oral contrast. To ensure treatment precision, image guidance was performed before each fraction using an electronic portal imaging device (EPID) or cone-beam computed tomography (CBCT).

**Fig. 2:**
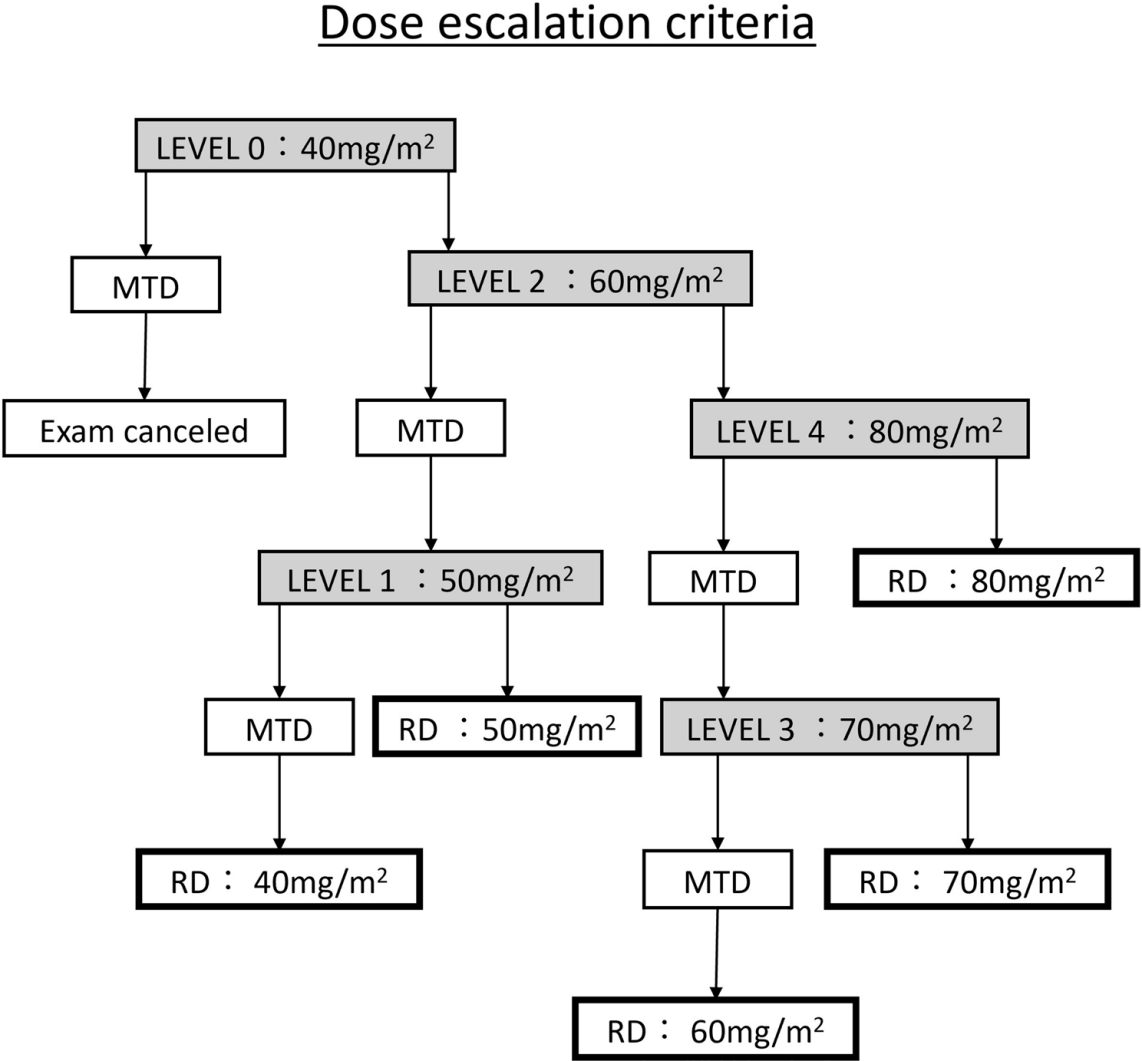
Dose escalation criteria. The dosage level was modified in increments of two when an increase was implemented, and in increments of one when a decrease was implemented

S-1 was administered orally twice daily on days 1 to 5 and 8 to 12 of a 14-day cycle, with the dose determined by body surface area. Irinotecan was administered intravenously on days 1 and 8. The initial dose of irinotecan was 40 mg/m² (Level 0). To optimize the identification of the maximum tolerated dose (MTD) while minimizing the number of patients treated at low, potentially sub-therapeutic doses, a modified 3 + 3 dose-escalation design was employed. This accelerated titration approach was justified by prior clinical experience at our institution, which demonstrated the safety of irinotecan at doses up to 60 mg/m² in combination with conventional long-course pelvic radiotherapy [8, 10]. In this protocol, dose levels were escalated by two increments if no dose-limiting toxicities (DLTs) were observed in the initial three-patient cohort. If one or two of the three patients experienced a DLT, the cohort was expanded to six patients. If fewer than 50% of patients (e.g., < 3 out of 6) in the expanded cohort experienced a DLT, two levels escalated the dose. The MTD was defined as the dose level at which DLT occurred in 50% of patients (i.e., at least 3 out of 6 patients). Once the MTD was identified, the dose was de-escalated by one level to confirm safety. If the MTD criteria were also met at this lower level, the next lower level was designated as the recommended dose (RD) (Fig. 2). This accelerated escalation strategy was adopted to improve the efficiency of dose-finding in the context of combined chemoradiotherapy, where the safety of low-dose irinotecan was anticipated based on prior clinical data.

The re-staging procedure was conducted within 12 to 16 weeks following the conclusion of CRT. This procedure involved CT, magnetic resonance imaging (MRI), and colonoscopy to assess treatment response and potential complications. No regulations were in place regarding treatment after chemoradiotherapy, with patients permitted to choose between consolidation chemotherapy or surgery after chemoradiotherapy. In the event of a complete clinical response (cCR), non-operative management (NOM) was authorized.

### Definition of DLT

The evaluation of adverse events was based on the Common Terminology Criteria for Adverse Events (CTCAE) version 5.0. DLT was defined as any of the following symptoms occurring by day 14 after the conclusion of chemoradiotherapy: (1) Grade 4 hematological toxicity (2), fever of 38 °C or higher associated with Grade 3 neutropenia (3), Grade 3 or higher thrombocytopenia (4), Grade 3 or higher non-hematological toxicity (excluding anorexia and nausea/vomiting) (5), reduction of the S-1 dose or fewer than 2/3 prescribed days of administration (i.e., fewer than four days) due to an adverse event (6), omission or reduction of irinotecan administration due to an adverse event, and (7) suspension of radiotherapy for one week due to an adverse event.

### The endpoint

The primary objective of the study was to estimate the maximum MTD and RD of irinotecan. The secondary endpoints encompassed adverse events associated with chemoradiotherapy, down-staging rate, histological treatment effect, pathological complete response (pCR) rate, and R0 resection rate. Histological response was assessed only in cases with surgery. The assessment was based on the following criteria in accordance with the 9th edition of the Japanese Classification of Colorectal, Appendiceal, and Anal Carcinoma: Grade 0 (no effect): It has been demonstrated that treatment does not result in the degeneration or necrosis of cancer cells. Grade 1a (mild effect): A study of the sample revealed that fewer than one-third of the cancer cells were degenerated or necrotic. Grade 1b (moderate effect): The process of degeneration, necrosis, or melting of cancer cells is observed in more than one-third and less than two-thirds of the cancerous tissue. Grade 2 (significant effect): A substantial degree of degeneration, necrosis, melting, or disappearance has been observed in more than two-thirds of the cancerous tissue. Grade 3 (significant effect): The neoplastic cells comprising the malignancy have undergone necrosis, undergone liquefaction, or other forms of cell death. The neoplasm has been replaced by granulomatous tissue or fibrotic foci [11].

## Results

### Patient characteristics

**Fig. 3:**
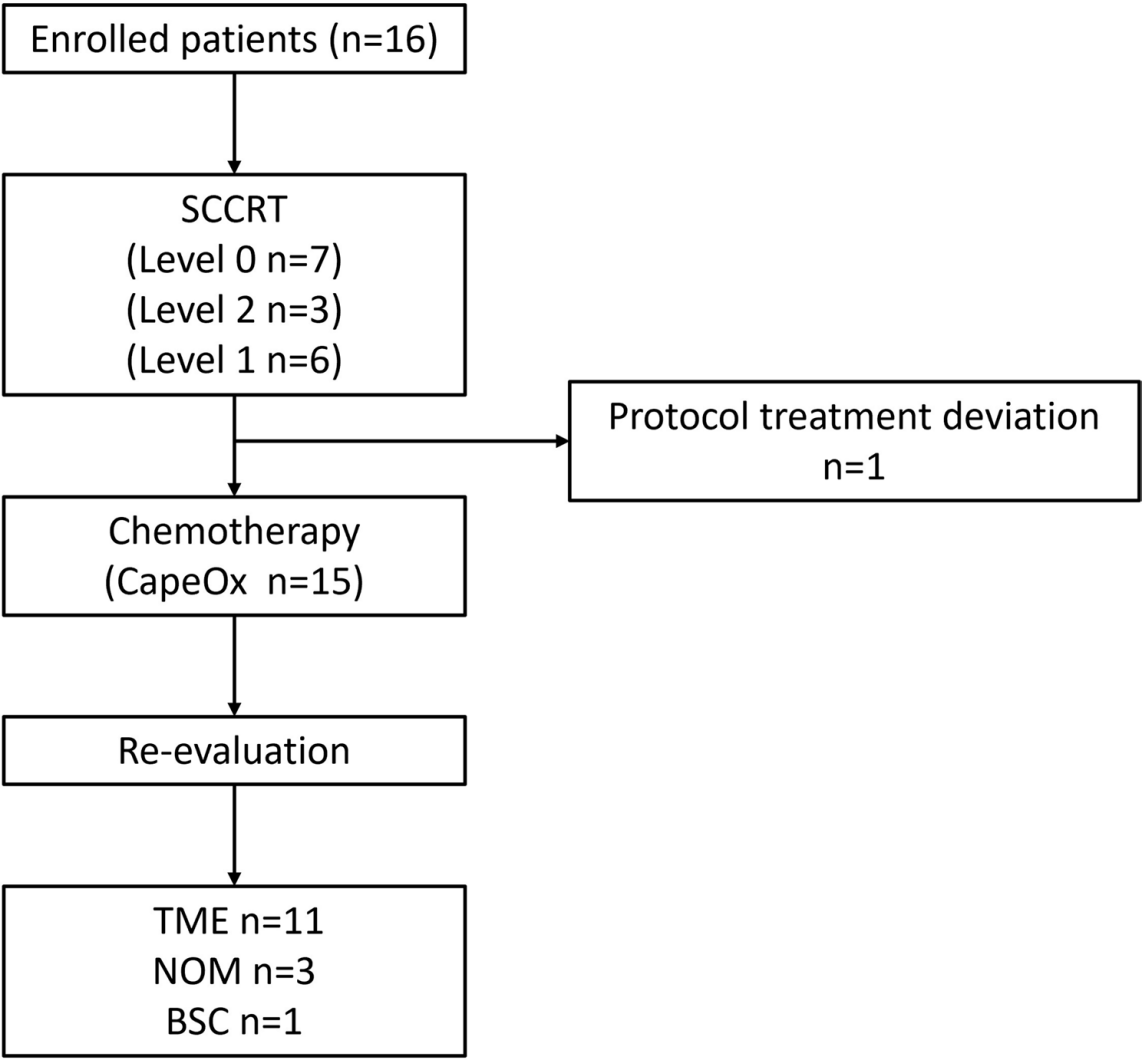
The patient enrollment diagram is presented below. One patient was excluded from the study during the treatment period due to inadequate compliance with S-1. A total of 15 patients were assessed. The remaining 15 patients underwent consolidation chemotherapy. Subsequent to chemotherapy, 11 patients underwent TME. NOM was performed on three patients. One patient received BSC. SCCRT, short-course chemoradiotherapy; TME, total mesorectal excision; NOM, non-operative management; BSC, best supportive care

The patient enrollment diagram is depicted in Fig. 3. From February 2022 to July 2024, a total of sixteen patients were enrolled in this study. However, one patient was excluded from the study due to noncompliance with the prescribed protocol during CRT. The patient characteristics are enumerated in Table 1.

**Table 1:** Patients’ characteristics

		Total (*n* = 15)	Level 0 (*n* = 6)	Level 2 (*n* = 3)	Level 1 (*n* = 6)
Dose (mg/m^2^)			40	60	50
Number of patients		9	6	3	9
Gender (%)					
	Female	6 (40)	3 (50.0)	0 (0)	3 (50.0)
	Male	9 (60)	3 (50.0)	3 (100)	3 (50.0)
Age (years)					
	Median	66	60.5	67	61
	Range	39–74	44–74	67–70	39–71
BMI (kg/m^2^)					
	Median	21.1	25.2	21.1	19.3
	Range	16.4–29.1	17.9–29.1	20.8–24.3	16.4–26
ASA (%)					
	Class I	15 (100)	6 (100)	3 (100)	6 (100)
	Class II	0 (0.0)	0 (0.0)	0 (0.0)	0 (0.0)
	Class III	0 (0.0)	0 (0.0)	0 (0.0)	0 (0.0)
Location (%)					
	Rab	4 (26.7)	1 (16.7)	0 (0.0)	3 (50.0)
	Rb	10 (66.7)	5 (83.3)	3 (100)	2 (33.3)
	Rbp	1 (6.6)	0 (0.0)	0 (0.0)	1 (16.7)
AV (cm)					
	Median	4.6	4.9	1.8	3.4
	Range	0–8	3–8	0-5.4	1.9-6
Diameter (mm)					
	Median	35.1	30.7	30.9	39.3
	Range	7-68.5	7-54.5	29.3–49.7	19.1–68.5
Differentiation (%)					
	pap	1 (6.7)	1 (16.7)	0 (0.0)	0 (0.0)
	tub1	8 (53.3)	1 (16.7)	2 (66.7)	5 (83.3)
	tub2	6 (40.0)	4 (66.7)	1 (33.3)	1 (16.7)
Circumference (%)					
	Median	75	41.5	75	75
	Range	20–100	20–75	75–80	25–75
cT (%)					
	3	12 (80)	4 (66.7)	3 (100)	5 (83.3)
	4a	3 (20)	2 (33.3)	0 (0.0)	1 (16.7)
cN (%)					
	0	10 (66.7)	4 (66.7)	3 (100)	3 (50%)
	1a	1 (6.7)	1 (16.7)	0 (0.0)	0 (0.0)
	1b	2 (13.3)	0 (0.0)	0 (0.0)	2 (33.3)
	2a	1 (6.7)	1 (16.7)	0 (0.0)	0 (0.0)
	2b	1 (6.7)	0 (0.0)	0 (0.0)	1 (16.7)
cStage (%)					
	IIa	9 (60.0)	3 (50.0)	3 (100)	3 (50.0)
	IIb	1 (6.7)	1 (16.7)	0 (0.0)	0 (0.0)
	IIIa	0 (0.0)	0 (0.0)	0 (0.0)	0 (0.0)
	IIIb	3 (20.0)	1 (16.7)	0 (0.0)	2 (33.3)
	IIIc	2 (13.3)	1 (16.7)	0 (0.0)	1 (16.7)

Patient characteristics. BMI, body mass index; ASA, American Society of Anesthesiologists Physical Status Classification System; AV, anal verge

### Levels of DLT and RD

All patients completed short-term chemoradiotherapy. No patients discontinued or withdrew from treatment. At Level 0 (40 mg/m^2^), one of the first three patients developed Grade 3 diarrhea, prompting enrollment of three additional patients. In the Level 2 cohort (60 mg/m^2^), all three patients experienced Grade 3 diarrhea. In the Level 1 cohort (50 mg/m^2^), one of the initial three patients experienced Grade 3 diarrhea, and three additional patients were enrolled. Of the three additional cases, only one patient exhibited Grade 3 diarrhea (Table 2). Among the six cases, DLT was only observed in two. Consequently, we defined 60 mg/m² as the MTD and 50 mg/m² as the RD of irinotecan. Table 3 provides detailed descriptions of adverse events. The occurrence of Grade 3 or higher adverse events was 40% (6 out of 15 cases), and at the recommended irinotecan dose, it was 33.3% (2 out of 6 cases).

**Table 2:** Number of patients with DLT

	Level 0 (*n* = 6)	Level 2 (*n* = 3)	Level 1 (*n* = 6)
Hematologic toxicity (≧G3)	0	0	0
Fever ≧ 38℃ with Neutropenia (≧G3)	0	0	0
Thrombocytopenia (≧G3)	0	0	0
Non-hematological toxicity (≧G3)	1 (16.7%)	3 (100%)	2 (33.3%)
S-1 dose reduction	0	0	0
irinotecan dose reduction	0	0	0
Suspension of RT	0	0	0

Dose limited toxicity (DLT). Any of the above symptoms that occur by day 28 after the end of chemoradiation therapy will be considered a DLT

**Table 3:** Details of adverse events

	Total		Level 0 (*n* = 6)		Level 2 (*n* = 3)		Level 1 (*n* = 6)	
	Any grade	Grade 3	Any grade	Grade 3	Any grade	Grade 3	Any grade	Grade 3
Leukopenia	0	0	0	0	0	0	0	0
Neutropenia	0	0	0	0	0	0	0	0
Thrombocytopenia	1	0	1	0	0	0	0	0
Bilirubin elevation	0	0	0	0	0	0	0	0
AST elevation	0	0	0	0	1	0	1	0
ALT elevation	5	0	1	0	2	0	2	0
ALP elevation	0	0	0	0	0	0	0	0
Creatinine elevation	2	0	1	0	1	0	0	0
Fever	2	0	1	0	1	0	0	0
HFS	0	0	0	0	0	0	0	0
Pigmentation	0	0	0	0	0	0	0	0
Alopecia	0	0	0	0	0	0	0	0
Rash	1	0	1	0	0	0	0	0
Constipation	2	0	2	0	0	0	0	0
Diarrhea	21	6	6	1	3	3	6	2
Stomatitis	3	0	1	0	0	0	2	0
Nausea	3	0	1	0	1	0	1	0
Vomiting	0	0	0	0	0	0	0	0
Appetite loss	6	0	3	0	1	0	2	0
Dehydration	0	0	0	0	0	0	0	0
Hypoalbuminemia	9	0	1	0	3	0	5	0
Anemia	12	0	6	0	3	0	3	0
Febrile neutropenia	0	0	0	0	0	0	0	0
Fatigue	1	0	1	0	0	0	0	0
Neuropathy	0	0	0	0	0	0	0	0
Dyspnea	0	0	0	0	0	0	0	0
Hypoxia	0	0	0	0	0	0	0	0
Pneumonia	0	0	0	0	0	0	0	0

AST, aspartate aminotransferase; ALT, alanine aminotransferase; ALP, alkaline phosphatase;

HFS, hand-foot syndrome

### Post-SCCRT treatment

CapeOX was administered after SCCRT for all patients. After chemotherapy, tumor re-staging was performed, and 3 of the 15 patients were determined to have achieved cCR or near CR. Subsequently, these patients underwent NOM. Surgery was performed on 11 of the 15 patients. One patient exhibited an incomplete response and was recommended for surgical intervention; however, the patient declined and received best supportive care (Table 4).

**Table 4:** Results of treatment after SCCRT

Patient no.	Level	cT	cN	Treatment after TNT	ypT	ypN	Down staging	Remnant tumor (*R*)	Primary tumor pathology
1	level 0	3	0	NOM	-	-	-	-	cCR
2	level 0	3	0	LAR	2	1a	no	0	Grade 2
3	level 0	4a	2a	LAR	3	0	yes	0	Grade1b
4	level 0	3	0	NOM	-		-	-	cCR
5	level 0	4a	0	LAR	pCR	0	yes	0	Grade 3
6	level 0	3	1a	NOM	-	-	-	-	cCR
7	level 2	3	0	BSC	-	-	-	-	iCR
8	level 2	3	0	LAR	2	0	yes	0	Grade 1b
9	level 2	3	0	APR	2	0	yes	0	Grade 2
10	level 1	3	0	APR	2	0	yes	0	Grade 1a
11	level 1	4a	2b	LAR	3	1b	yes	0	Grade1b
12	level 1	3	0	LAR	pCR	0	yes	0	Grade 3
13	level 1	3	1b	LAR	3	0	yes	0	Grade 2
14	level 1	3	1b	LAR	pCR	0	yes	0	Grade 3
15	level 1	3	0	LAR	1b	0	yes	0	Grade 1a

Results of treatment after SCCRT. ypT and yp N indicate pathological stage after neoadjuvant therapy. TNT, total neoadjuvant therapy; NOM, non-operative management; LAR, Low anterior resection; BSC, best supportive care; APR, Abdominoperineal resection; cCR, complete clinical response; iCR, incomplete response

### Operative results

The surgical procedures involved nine low anterior resections and two abdominoperineal resections. The anal sphincter preservation rate was 81.8%. Among patients with RD or lower irinotecan dose levels, nine underwent surgery, and none experienced Grade 3 or higher complications according to the Clavien-Dindo classification.

### Pathological results

R0 resection was achieved in all surgical cases. Pathologic downstaging occurred in 10 of 11 cases (90.9%). Moreover, a pathological complete response was achieved in three cases (27.3%). The details of the therapeutic effect grade are shown in Table 4.

## Discussion

The clinical management of locally advanced rectal cancer (LARC) has shifted toward Total Neoadjuvant Therapy (TNT) to improve systemic control and organ preservation. This phase I study established the recommended dose (RD) of irinotecan at 50 mg/m² in a novel SCCRT regimen with S-1 and irinotecan. Crucially, this study provides the first safety evidence for integrating a potent S-1/irinotecan doublet with short-course radiotherapy (SCRT), offering a potential alternative to conventional long-course chemoradiotherapy (LCRT).

Recent landmark RCTs have redefined the TNT landscape, yet the optimal regimen remains a subject of intense debate. The RAPIDO and STELLAR trials established the efficacy of SCRT followed by consolidation doublet chemotherapy (CapeOX or FOLFOX). While these trials confirmed improved disease-related treatment failure rates (DrTF) or Disease Free Survival (DFS), they primarily relied on radiotherapy alone for local control [5, 6]. In contrast, our regimen incorporates S-1 and irinotecan concurrently with SCRT. By leveraging the dual radio sensitizing effects of dehydropyrimidine dehydrogenase (DPD) inhibition (via S-1) [12] and Topoisomerase I **(**TOP1) inhibition (via irinotecan) [13], our approach aims to maximize the biological intensity of the pre-operative treatment from the outset. This intensified concurrent chemotherapeutic backbone during the radiation phase may offer superior downstaging compared to the standard SCRT-only or SCRT plus doublet approach used in RAPIDO. The OPRA trial utilized long-course chemoradiotherapy (LCRT) to maximize the chances of a clinical complete response (cCR) for organ preservation. While LCRT is effective, it requires 5.5 weeks of daily hospital visits [14]. Our SCCRT-based TNT provides a logistically efficient 1-week radiation phase without necessarily sacrificing local potency. The 27% pCR rate observed in our small cohort, even at the dose-finding stage, suggests that this intensified SCCRT could be a viable, time-efficient alternative for achieving cCR and facilitating watch-and-wait strategies.

The synergistic mechanism of S-1 and irinotecan as radiosensitizers is central to this protocol. Irinotecan-mediated TOP1 inhibition prevents the repair of DNA double-strand breaks induced by 5 Gy fractions of SCRT [15]. Interestingly, our RD of 50 mg/m² is lower than the 60 mg/m² typically used in LCRT (the SAMRAI trial) [8]. This disparity suggests a fractionation-dependent toxicity profile, in which the higher daily dose of 5 Gy may sensitize the intestinal mucosa to irinotecan, necessitating the specific dose-calibrations established in this study [16, 17].

Although the results are encouraging, several limitations should be recognized. Firstly, since this was a phase I trial conducted at a single institution, it involved a small sample size (*n* = 15), limiting the wider applicability of our safety and efficacy results. The limited number of participants also prevents a conclusive evaluation of long-term cancer outcomes. Secondly, the primary focus was on acute toxicities and DLTs within a 14-day window. Consequently, late toxicities, including long-term functional outcomes (anorectal or sexual dysfunction) and late bowel toxicity, remain to be evaluated. Thirdly, there is a potential for selection bias, particularly regarding patients who opted for non-operative management (NOM) after achieving cCR. The criteria for selecting NOM versus surgery were not randomized, which may have influenced the reported pCR and cCR rates. Additionally, although consolidation CapeOX was planned for all patients, compliance varied, which may confound the assessment of the total TNT effect versus the specific contribution of the SCCRT phase.

## Conclusion

Despite these limitations, this study demonstrates that SCCRT with S-1 and irinotecan (RD: 50 mg/m²) is a feasible and potent component of a TNT strategy. It bridges the gap between the logistical convenience of SCRT and the high local intensity of triplet-based chemotherapy. Given these encouraging results, this SCCRT protocol will be further evaluated in the context of Total Neoadjuvant Therapy (TNT), followed by consolidation chemotherapy in the upcoming SHOWTIME study. This transition has the potential to optimize oncological outcomes while significantly reducing the medical and logistical burden on both patients and healthcare systems.

## Supplementary Information

Below is the link to the electronic supplementary material.

Supplementary Material 1

## Data Availability

The datasets generated and/or analysed during the current study are available from the corresponding author on reasonable request.

## Abbreviations

TNT: Total neoadjuvant therapy
SCCRT: Short course chemoradiotherapy
SCRT: Short course radiotherapy
MTD: Maximum tolerated dose
RD: Recommend dose
DLT: Dose-limiting toxicity
CRT: Chemoradio therapy
TME: Total mesorectum excision
DrTF: Disease-related treatment failure
DFS: Disease-free survival
UICC: Union for International Cancer Control
ECOG- PS: Eastern Cooperative Oncology Group Performance status
GTV: Gross target volume
CTV: Clinical target volume
PTV: Planning target volume
CT: Computed tomography
MRI: Magnetic resonance imaging
NOM: Non-operative management
cCR: Complete Clinical response
CTCAE: Common Terminology Criteria for Adverse Events
pCR: Pathological complete response
iCR: Incomplete response
RCTs: Randomized controlled trials
CDHP: 5-chloro-2,4-dihydroxypyridine
DPD: Dihydropyrimidine dehydrogenase
TOP1: Type I topoisomerase
