# Supplementary material for: The phase I trial of preoperative short-course chemoradiotherapy with S-1/irinotecan and short-course radiation in locally advanced lower rectal cancer (SHOWTIME study)
Source: Radiat Oncol. 2026 Jun 10;21:109. doi: 10.1186/s13014-026-02872-3 (PMC13480079; doi:10.1186/s13014-026-02872-3)
Supplement: Supplementary file 1 [file 13014_2026_2872_MOESM1_ESM.docx]

**Supplement. 1**

**Patient selection criteria**

**Eligibility criteria**

1. Cases confirmed histologically as rectal cancer (adenocarcinoma)
2. Cases that meet all of the following criteria based on imaging diagnosis* within 28 days before registration:

- Resectable cases diagnosed as cT3-4 and N0-2
- • The primary tumor site is either Ra or Rb.
- • The lower edge of the tumor is located on the anal side of the peritoneal reflection.
- • There is no distinct lateral lymph node metastasis.
- • The tumor is not type 4 (diffuse infiltrating type) according to the macroscopic classification.

* Diagnosis by MDCT and MRI is required. However, simple imaging is acceptable in cases of contrast agent allergy. Furthermore, EUS may be used as an adjunct to exclude T1-2 cases.

1. Cases without liver, peritoneal, or distant metastasis
2. Cases with a registered age of 20 or older and 80 or younger (full age)
3. Cases without prior treatment (radiation therapy, chemotherapy, hormone therapy, etc.) for the target disease
4. Cases without severe impairment of major organ (bone marrow, liver, kidney, etc.) function

(All of the following must be achieved in tests conducted within 14 days prior to registration)

- White blood cell count: 4,000/mm3 or more and less than 12,000/mm3
- Neutrophil count: 2,000/mm3 or more
- Platelet count: 100,000/mm³ or higher
- Hemoglobin: 9.0 g/dl or higher
- Total bilirubin: 1.5 mg/dl or higher
- AST (GOT) and ALT (GPT): 100 IU/L or lower
- Serum creatinine: 1.2 mg/dl or lower
- Estimated creatinine clearance: 60 ml/min or higher (Calculated using the Cockcroft-Gault formula***) 【Cockcroft-Gault formula】Male Ccr = weight (kg) × (140 - age) / (72 × serum creatinine level), Female Ccr = Male Ccr × 0.85

1. Cases with no clinically significant findings on an electrocardiogram obtained within 28 days prior to registration
2. Cases with a general condition of ECOG-Performance Status (P.S.) 0-1
3. Cases capable of eating and receiving oral medication
4. Cases in which the subject has been informed of the details of the present study using an informed consent form and has provided written consent

**Exclusion criteria**

1. Cases with a history of severe drug hypersensitivity
2. Cases with active concurrent cancer or multiple colorectal cancers. Active concurrent cancer refers to synchronous concurrent cancer and metachronous concurrent cancer with a disease-free interval of 5 years or less. However, carcinoma in situ (epithelial carcinoma), adenocarcinoma of gastric cancer, and skin cancer that are considered cured by treatment are not included in active concurrent cancer. Furthermore, colon cancer is not included in multiple colon cancers.
3. Cases receiving flucytosine or atazanavir sulfate
4. Cases with UGT1A1*6/*6, UGT1A1*28/*28, or both heterozygous (UGT1A1*6/*28)
5. Cases with active infections (fever of 38.0°C or higher)
6. Cases with severe complications (intestinal paralysis, intestinal obstruction, interstitial pneumonia or pulmonary fibrosis, uncontrolled diabetes, uncontrolled hypertension, heart failure, renal failure, hepatic failure, etc.)
7. Cases with a history of interstitial pneumonia
8. Cases with pleural effusion or ascites requiring treatment
9. Cases with recent gastrointestinal bleeding
10. Cases with diarrhea (watery stools)
11. Women who are pregnant or may be pregnant (as determined by a physician), pregnant women, or nursing mothers. Cases with no intention of using contraception
12. Cases receiving continuous systemic steroid administration (oral or intravenous)
13. Cases that are HBs antigen-positive
14. Cases in which administration of S-1, CPT-11, capecitabine, or L-OHP is contraindicated
15. Other cases the attending physician deems inappropriate for enrollment in the present study

**Discontinuation criteria for each study subject**

The study will be discontinued if any of the following criteria are met.

1) If the study subject withdraws their consent.

2) If compliance with the study protocol becomes impossible.

3) If the study is discontinued.

4) Other cases in which the principal investigator or sub-investigator determines that continuing the study is difficult.
